# Supplementary material for: Smartphone Addiction and Related Factors among Athletes
Source: Behav Sci (Basel). 2024 Apr 18;14(4):341. doi: 10.3390/bs14040341 (PMC11154377; doi:10.3390/bs14040341)
Supplement: Supplementary file 1 [file behavsci-14-00341-s001.zip › behavsci-2889071-supplementary.pdf]

Supplementary Table S1

Number of Athletes in Handball Teams

Name of Sports Club	Professional Women's Team (Number of Athletes)	Professional Men's Team (Number of Athletes)	Licensed Women's Youth Teams (Number of Athletes) (Youth)	Licensed Men's Youth Teams (Number of Athletes) (Youth)
İzmir B.Ş.B.S.K.	16	16	12	12
Göztepe S.K.	X	16	X	12
İzmir G.H.S.İ.M. S.K.	16	X	14	X
Bornova Bld. S.K.	16	X	14	X
1970 Vefa G.S.K.	14	X	X	X
Gaziemir Atletizm S.K.	X	12	X	X
Dikili Günes S.K.	X	14	X	X
Seferihisar Cözum Koleji S.K.	X	16	X	X
A.T.S.K.	X	12	X	X
Total	62	85	40	24
	212			

Supplementary Table S2

Mean, SD, Deviation, etc. of Data Related to TFEQ and Body Perception

Data Group	Variables	N	Minimum	Maximum	Mean	Std. Deviation
Eating Status	Number of Main Meals per Day	202	1	5	2.58	0.70
	Number of Snacks per Day	202	0	6	1.86	1.07
	TFEQ scale total score	202	18	71	47.66	11.07
	Inability to restrain eating	202	5	20	13.15	3.69
	Emotional Eating	202	3	12	8.38	2.94
	Conscious restriction of eating	202	6	23	14.51	3.81
	Hunger	202	4	16	11.60	3.82
Body Perception	Appreciation of Own Body Structure (1-4)	202	1	4	2.26	0.68
	Media Interest in Ideal Body Structure (1-3)	202	1	3	1.88	0.83
	Body Perception Scale Score	202	40	192	87.20	29.40
